# Diagnostic and Prognostic Significances of MUC5B and TTF-1 Expressions in Resected Non-Small Cell Lung Cancer

**DOI:** 10.1038/srep08649

**Published:** 2015-03-03

**Authors:** Ryo Nagashio, Junpei Ueda, Shinichiro Ryuge, Hiroyasu Nakashima, Shi-Xu Jiang, Makoto Kobayashi, Kengo Yanagita, Ken Katono, Yukitoshi Satoh, Noriyuki Masuda, Yoshiki Murakumo, Kazuo Hachimura, Yuichi Sato

**Affiliations:** 1 Department of Applied Tumor Pathology, Graduate School of Medical Sciences, Kitasato University, Kanagawa, Japan; 2 Department of Molecular Diagnostics, School of Allied Health Sciences, Kitasato University, Kanagawa, Japan; 3 Department of Respiratory Medicine, School of Medicine, Kitasato University, Kanagawa, Japan; 4 Department of Thoracic and Cardiovascular Surgery, School of Medicine, Kitasato University, Kanagawa, Japan; 5 Department of Pathology, School of Medicine, Kitasato University, Kanagawa, Japan

## Abstract

To investigate the relationships between the expression of MUC5B and clinicopathological parameters, the expression of MUC5B was immunohistochemically studied. MUC5B expression was observed in 129 of 198 (65.2%) adenocarcinomas and in 4 of 49 (8.2%) squamous cell carcinomas (P < 0.00001). MUC5B expression was significantly associated with poorer differentiation (P = 0.0303), higher pathological TNM stage (p = 0.0153) and poorer prognosis of adenocarcinoma patients (P = 0.0017). Multivariable analysis with Cox proportional hazards models confirmed that MUC5B expression increased the hazard of death after adjusting for other clinicopathological factors (HR = 2.66; 95%CI, 1.26–5.61). We also immunohistochemically evaluated TTF-1 expression and found that the combination of MUC5B with TTF-1 is a useful marker for adenocarcinomas. The diagnostic accuracies of TTF-1 and MUC5B for adenocarcinoma were 83.8% and 70.4%, respectively. The accuracy increased to 94.3% when the two factors were combined. In survival analysis, the MUC5B(High)/TTF-1(−) group was significantly associated with a poorer outcome compared with the MUC5B(Low)/TTF-1(+) group (p < 0.0001). The present study suggested that the combination of MUC5B and TTF-1 expression is useful for discriminating adenocarcinomas from squamous cell carcinomas, yielding prognostic significance in patients with lung adenocarcinoma.

Primary lung cancer is the leading cause of cancer death, and the percentage of adenocarcinoma (AC) among lung cancers has been increasing gradually in recent decades^1^,^2^. While surgical resection is the optimal treatment for early-stage non-small cell lung cancer (NSCLC), the 5-year survival rates for surgically resectable NSCLC are still unsatisfactory and range from 19% for stage IIIA to 63% for stage IA^2^. Recent advances in molecular biology have raised the possibility of new treatments for NSCLCs, such as tailor-made chemotherapy based on biomarkers or molecular-targeted agents^3^,^4^. For ACs, molecular-targeted therapies against vascular endothelial growth factor and epidermal growth factor receptor have been used. However, avastin (bevacizumab) is contraindicated in patients with squamous cell carcinoma (SCC) because about 30% of patients die from fatal hemoptysis^5^,^6^. Therefore, it is necessary to research effective methods to accurately discriminate between AC and SCC, and thereby inform the selection of appropriate therapies in NSCLCs.

Antibodies are usually developed using purified proteins or synthetic peptides. We have exhaustively generated monoclonal antibodies (MoAbs) against various tumor-associated proteins using lung cancer cell lines or tissues as antigens with the random immunization method^7^, and have obtained over 2,000 MoAbs^8^,^9^. This method is expected to generate antibodies against proteins with tumor-specific post-translational modifications that are difficult to obtain by conventional immunization methods. The present study describes one such antibody, KU-Lu-7, which reacted with bronchial epithelial cells with mucin, and was frequently highly expressed in lung ACs. By immunoprecipitation and mass spectrometry, it was confirmed that the KU-Lu-7 antibody recognizes MUC5B (Supplementary figure 1).

Mucins are high molecular weight O-glycosylated proteins and are present in most epithelial cells. Human mucins are structurally classified into two families, membrane-bound mucins and secreted or gel-forming/polymerizing mucins, and MUC5B belongs to the latter^10^,^11^. MUC5B has a critical protective function in the normal lung, salivary glands, esophagus and gallbladder, and has been reported to be aberrantly expressed in breast cancer^12^. Although a few studies have focused on MUC5B in lung cancers, no report has detailed the relationships between MUC5B expression and clinicopathological features in NSCLC. Moreover, it has been reported that MUC5B is a target gene of TTF-1, which is involved in lung development and carcinogenesis, and strongly represses MUC5B expression^13^. Although TTF-1 is well known as a useful marker for lung ACs, it is also reported that no or low TTF-1 expression is detected in mucinous ACs. Because these ACs may express MUC5B, the diagnostic accuracy of lung AC should be increased by immunostaining with both of these factors. Therefore, the objectives of this study were: (1) to immunohistochemically examine MUC5B expression in tumor cells of 198 ACs and 49 SCCs, (2) to evaluate the relationships between MUC5B expression in tumor cells and the clinicopathological parameters of ACs, and (3) to estimate the diagnostic accuracy of combined MUC5B and TTF-1 expressions in ACs.

## Results

### Patient Characteristics

The clinicopathological characteristics of the patients are summarized in Table 1. In total, 154 male and 93 female patients were included with ages ranging from 34 to 82 years (median, 65 years), of whom 151 (61.1%) were smokers. There were 147 (59.5%) stage I (100 stage IA and 47 stage IB), 48 (19.4%) stage II (25 stage IIA and 23 stage IIB), and 52 (21.1%) stage III (49 stage IIIA and 3 stage IIIB) diseases, including 198 (80.2%) ACs and 49 (19.8%) SCCs. Thirty-seven (15.0%) of the patients received adjuvant chemotherapy. The overall follow-up durations ranged from 3 to 127 months (median, 85 months). A total of 146 patients were alive at the end of the follow-up, while 76 patients died of lung cancer, 17 patients died from other causes, and 8 patients were lost to follow-up.

### MUC5B Expression in ACs and SCCs

**Figure 1:**
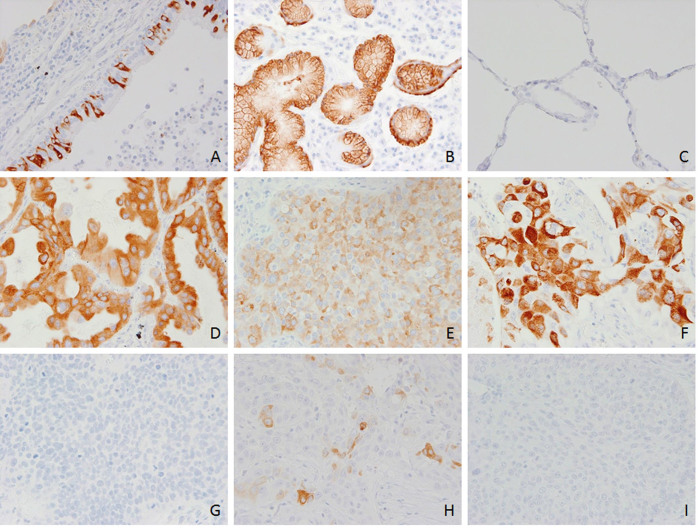
Expressions of MUC5B in normal lung tissues and lung cancers. Scattered cells with moderate to strong expression of MUC5B in bronchial epithelial cells (A). Strong staining was also found in bronchial glands (B). No obvious staining was observed in alveolar epithelial cells (C). Various extents of MUC5B expression in the cytoplasm of lung adenocarcinomas (D = well differentiated; E = moderately differentiated; F = poorly differentiated). The staining was almost completely absent in squamous cell carcinomas (G = well differentiated; H = moderately differentiated; I = poorly differentiated).

The expression of MUC5B was localized in the cytoplasm of tumor cells and was observed in 133 of 247 ACs and SCCs (53.8%)(Figure 1). Scattered positive cells were also constantly observed in the bronchial mucosa, which served as an internal control (Figure 1). They were further divided into 129 of 198 (65.2%) ACs and 4 of 49 (8.2%) SCCs and their mean staining scores of MUC5B were 4.2 and 0.3, respectively. The mean staining score in AC was significantly higher than that in SCC (p < 0.00001).

### Relationship between MUC5B Expression and Clinicopathological Characteristics in lung ACs

The relationships between MUC5B expression and clinicopathological characteristics in ACs are summarized in Table 2. MUC5B expression was related to poorer differentiation (P = 0.0303) and higher pathological TNM (p-TNM) stage (stage II and III) (P = 0.0153). Although it was not significant, there was a tendency toward higher MUC5B staining in the tumors with worse status for tumor size, nodal status, and pleural invasion. There were no significant associations between MUC5B expression and age, gender, smoking habit, vascular invasion, lymphatic invasion or adjuvant chemotherapy.

### Kaplan-Meier Estimate of Survival of AC Patients with high and low MUC5B Expression

**Figure 2:**
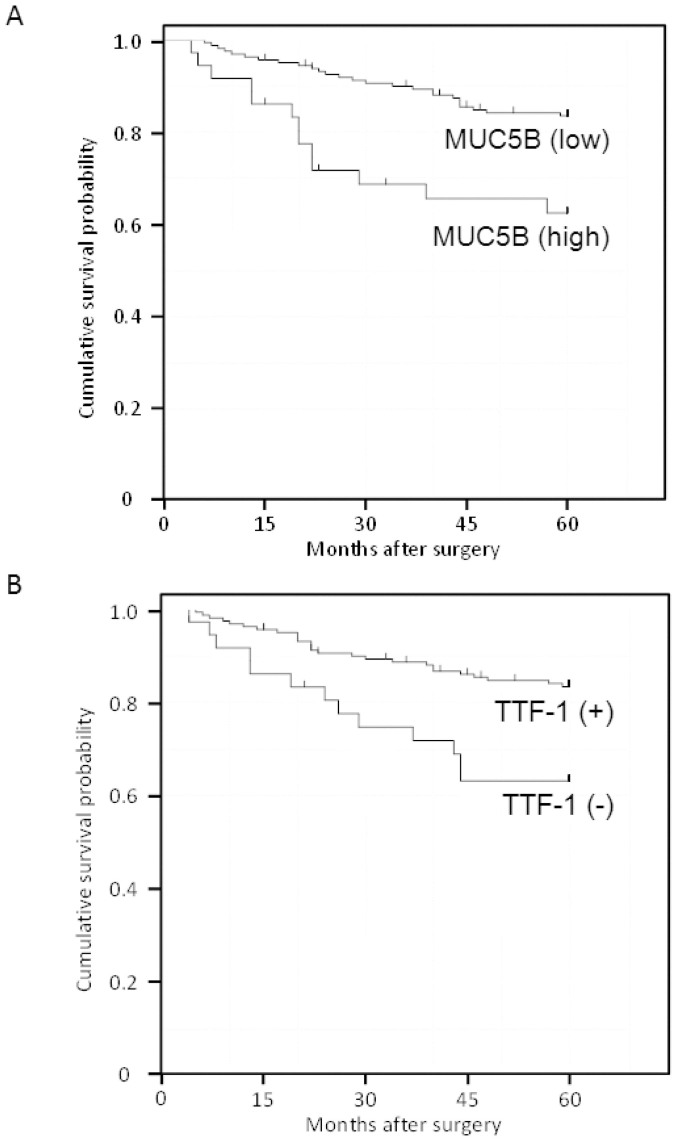
Cumulative survival of patients with lung adenocarcinoma according to MUC5B scores (High = score ≧ 9, Low = score < 9) (A) and TTF-1 expression (B) estimated by the Kaplan-Meier method, treating lost to follow-up as censored cases. MUC5B high expression group was significantly associated with poorer survival and TTF-1 expression was significantly associated with poorer survival.

All the patients were included in the survival analysis. The overall follow-up periods ranged from 3 to 127 months (median, 85 months). The mean survival time was 50 months, corresponding to a 5-year follow-up. Because a cumulative survival probability of 2.5% was not reached by the end of the 5-year follow-up, the overall median survival time was not determined. We divided the patients into two groups at score 9, which had the highest discrimination power. Five-year cumulative survival probability was 63.9% for the MUC5B-high expression group (score ≥ 9) and 84.0% for the MUC5B-low expression group (score < 9), and the difference was significant (P = 0.0017, Figure 2A).

### Effect of MUC5B Expression on Survival with Multivariable Analysis

The Cox proportional hazards model was applied to estimate the effect of MUC5B expression on survival. The crude hazard ratio (HR) of the MUC5B high expression group (score ≥ 9) compared to the MUC5B low expression group (score < 9) was 2.658 (95% CI, 1.260–5.608; P = 0.0102), which indicated that MUC5B-high expression status increased the hazard of lung cancer-related death. With multivariable analysis, MUC5B expression, tumor size, p-TNM stage, adjuvant chemotherapy, and vascular invasion were revealed to be significantly associated with survival (Table 3).

### TTF-1 Expression in ACs and SCCs

The expression of TTF-1 was localized in the nucleus of tumor cells and in normal alveolar epithelial cells; therefore, the stainability of the latter was used as an internal control. Nuclear TTF-1 expression in tumor cells was observed in 164 of 247 (66.4%) ACs and SCCs, including 161 of 198 (81.3%) ACs and 3 of 49 (6.1%) SCCs. The expression of TTF-1 was significantly higher in AC than in SCC (p < 0.00001, χ^2^ test).

### Relationship between TTF-1 Expression and Clinicopathological Characteristics in lung ACs

The relationships between TTF-1 expression and clinicopathological characteristics in ACs are summarized in Table 4. TTF-1 expression was inversely related to smoking habit (P = 0.0025), tumor size (P < 0.0001), tumor differentiation (P = 0.0011), and p-TNM stage (P = 0.0051), whereas there was no significant association between TTF-1 expression and age, gender, nodal status, vascular invasion, lymphatic invasion, pleural invasion or adjuvant chemotherapy.

### Kaplan-Meier Estimate of Five-year Survival of TTF-1-Positive and TTF-1-Negative AC patients

Five-year cumulative survival probability was 83.9% for the TTF-1-positive group and 64.9% for the TTF-1-negative group, and the difference was significant (P = 0.0044, Figure 2B).

### Evaluation of the diagnostic accuracy of MUC5B in combination with TTF-1 for lung ACs

**Figure 3:**
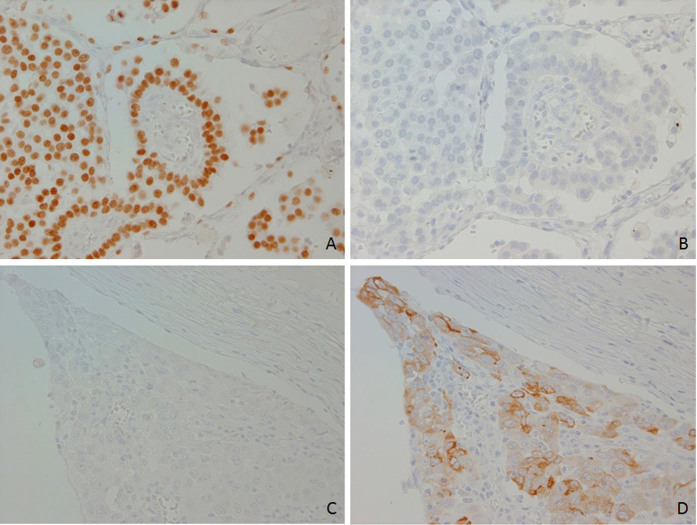
Lung adenocarcinoma cases without coexpression of MUC5B and TTF-1. A tumor was TTF-1 positive (A) and MUC5B negative (B), and a tumor was TTF-1 negative (D) and MUC5B positive (C) on serial sections.

To investigate the association between the expressions of MUC5B and TTF-1, we evaluated the stainability of MUC5B and TTF-1 in the same cases (Figure 3). While both expressions were coincidently recognized in about half of the cases, there were 29 MUC5B(+)/TTF-1(−) and 61 MUC5B(−)/TTF-1(+) tumors (Figure 3).

The diagnostic accuracy of TTF-1 or MUC5B for AC was 83.8% (81.3% sensitivity, 93.9% specificity) or 70.4% (65.2% sensitivity, 91.8% specificity). However, the accuracy of combined MUC5B and TTF-1 was higher, at 94.3% (96.0% sensitivity, 87.8% specificity).

### Kaplan-Meier Estimate of Survival for AC Patients by combining MUC5B and TTF-1 expression

**Figure 4:**
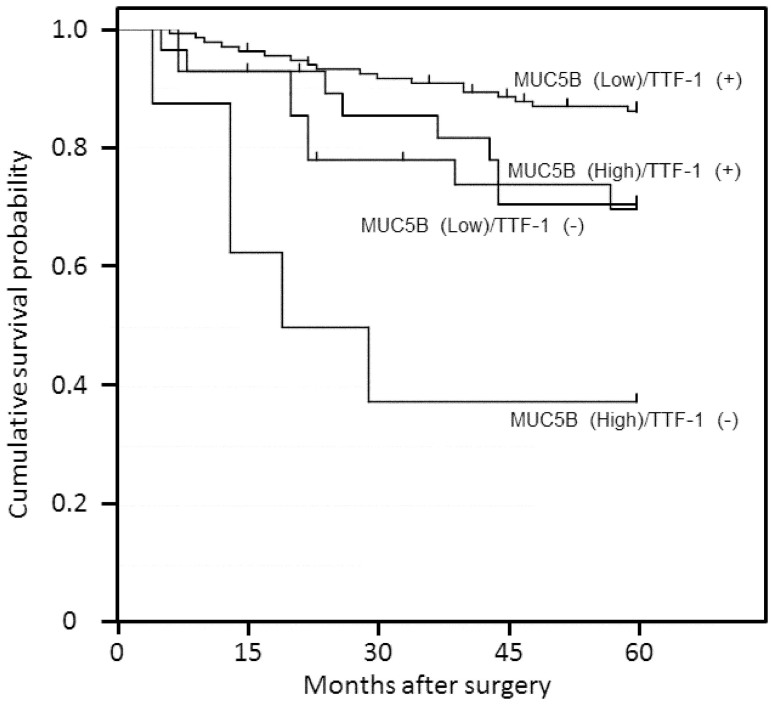
Cumulative survival of patients with lung adenocarcinoma according to MUC5B scores (High = score ≥ 9, Low = score < 9) combined with TTF-1 expression estimated by the Kaplan scores (High = score in the same part using the serial sections. The MUC5B High and TTF-1 negative group was significantly associated with poorer survival in patients with lung adenocarcinoma.

Figure 4 shows the five-year survival rate in the four groups separated by the expression of MUC5B and TTF-1, including 133 MUC5B(Low)/TTF-1(+), 28 MUC5B(High)/TTF-1(+), 29 MUC5B(Low)/TTF-1(−) and 8 MUC5B(High)/TTF-1(−). The five-year survival rates for the MUC5B(Low)/TTF-1(+), MUC5B(High)/TTF-1(+), MUC5B(Low)/TTF-1(−) and MUC5B(High)/TTF-1(−) groups were 86.5%, 71.4%, 72.4% and 37.5%, respectively. The MUC5B(High)/TTF-1(−) group had a significantly poorer outcome compared with the MUC5B(Low)/TTF-1(+) group (p < 0.0001), MUC5B(High)/TTF-1(+) group (p = 0.0423), and MUC5B(Low)/TTF-1(−) (p = 0.0308) group.

## Discussion

In this study, to identify useful differential diagnostic markers for lung ACs, we generated monoclonal antibodies using AMeX-fixed AC tissue as an immunogen by employing the random immunization method. One of the obtained monoclonal antibodies, KU-Lu-7, recognized MUC5B as confirmed by the combination of immunoprecipitation and mass spectrometry.

MUC5B is a secreted protein of molecular weight 596,340 Da with 5762 amino acids and belongs to the mucin family, which are highly glycosylated macromolecular components of mucus secretions and the major gel-forming mucin in mucus. Under physiological conditions, mucins play a protective role in epithelial tissues and are also involved in the processes of epithelial differentiation, growth regulation, modulation of cell adhesion, and cell signaling^14^. Overexpression of mucins is observed in many cancers^14^. Numerous studies have shown that abnormal mucin glycosylation is generally associated with a malignant transformation of epithelial cells^15^,^16^. Among the secreted mucins, MUC5B secretion is abnormally augmented in many airway diseases, such as chronic bronchitis, chronic obstructive pulmonary disease, asthma and cystic fibrosis^17^. MUC5B is also abnormally expressed in gastric and breast cancers and lung ACs^12^,^18^,^19^,^20^. To evaluate the utility of KU-Lu-7 antibody in lung cancer, we immunohistochemically studied 247 consecutive cases. Although the expression of MUC5B tended to be lower or absent in non-mucinous ACs, the staining score and the positivity rate were significantly higher in ACs compared with SCCs (P < 0.00001). Moreover, MUC5B expression was significantly associated with poorer differentiation (P = 0.0303) and p-TNM stage (p = 0.0153) of ACs. Copin et al. reported that non-mucinous and poorly differentiated ACs lost MUC5B expression^21^. MUC5B expression, however, was highly frequent in poorly differentiated ACs in the present study. This discrepancy may be due to the small number of cases (n = 34), and might be explained by the fact that majority of our well/moderately differentiated ones had more mature natures of Clara cell or pneumocytes but decreased mucus-producing ability. In this study, although it was not statistically significant, there was a tendency for higher MUC5B expression in tumors with worse status for tumor size, nodal status, and pleural invasion. These results suggest that MUC5B alone is not only a useful differential diagnostic marker of ACs from other histologic types of lung cancer, especially from SCCs, but also is a useful marker for more aggressive ACs.

Further, MUC5B expression was significantly associated with poorer survival (P = 0.0017). Multivariable analysis confirmed that MUC5B expression increased the hazard of death after adjusting for other clinicopathological factors (HR = 2.66; 95% CI, 1.26–5.61). Although Yu et al.^20^ reported that overexpression of MUC5 genes is associated with early post-operative metastasis in NSCLC, they did not study the detailed relationships between MUC5B expression and clinicopathological features such as tumor differentiation and p-TNM stage with a large number of NSCLC cases. Moreover, they performed northern-blot and slot-blot analyses using frozen bulk tissues including normal bronchial epithelium and stroma, and did not evaluate using only NSCLC cells. Consequently, 4 of 49 (8.2%) SCCs were weakly positive in the present study, whereas 6 of 11 (54.5%) MUC5B-overexpressed NSCLC cases were SCCs in their results. Their data may not reflect the influence of tumor cells. Zhang et al. reported that mucin production including MUC5B induced by AQP5 expression by gene transduction may play important roles in enhanced metastasis potential in lung AC cell lines^22^. Valque et al. also reported that MUC5B overexpression in MCF7 breast cancer cells by transfection with a vector encoding a recombinant mini-mucin MUC5B may enhance the aggressive behavior of tumor cells by increasing cell proliferation, tumor growth, and dissemination^23^. These findings suggest that MUC5B expression may be involved in more aggressive behavior of tumor cells, and MUC5B high expression seems to be an independent and significant predictor of poorer survival of lung AC patients.

Recently, many studies have focused on the chemoresistance of cancer. Advances in the field of molecular biology have contributed to the elucidation of chemoresistance mechanisms. Trials for the individualization of treatments, so-called tailor-made therapies, are one of these challenges. Accurate differential diagnosis is therefore fundamentally important.

TTF-1 is a homeodomain-containing nuclear transcription protein of the NKX2 gene family. It plays a critical role in the development and differentiation of bronchioalveolar cells through the activation of lung-specific genes coding surfactant and Clara cell secretory proteins^24^,^25^, and thus is a lineage marker of the terminal respiratory unit. In the adult lung, TTF-1 is expressed in type II pneumocytes, Clara cells and bronchiolar basal cells^26^. Because TTF-1 is commonly expressed in lung ACs, but not in SCCs, it is regarded as useful in the differential diagnosis of these two malignancies^27^. Martins reported that the expression of TTF-1 is an independent prognostic factor of lung AC by multivariable Cox proportional hazards regression analysis^28^. Meanwhile, MUC5B is a target gene of TTF-1, which acts as a strong repressor of MUC5B^13^, along with the finding that both expression were coincidently recognized in about half of the cases. Thus we can evaluate the effectiveness of the combination of MUC5B and TTF-1 in improving the accuracy of lung AC diagnosis. The accuracy of AC diagnosis using TTF-1 or MUC5B alone was 83.8% or 70.4%, respectively. However, the accuracy of the combination of MUC5B with TTF-1 was drastically increased, to 94.3%. In survival analysis, the MUC5B(High)/TTF-1(−) group had significantly poorer outcomes compared with the MUC5B(Low)/TTF-1(+) group (p < 0.0001), MUC5B(High)/TTF-1(+) group (p = 0.423), and MUC5B(Low)/TTF-1(−) (p = 0.0308) group. The present study suggests that the combination of MUC5B and TTF-1 is useful for discriminating ACs from SCCs, and is a prognostic indicator of survival probability of lung AC patients.

## Methods

### Patients and Tissue Specimens

Ten percent formalin-fixed and paraffin-embedded tissues of 247 consecutive ACs and SCCs with complete resection from January 2002 to September 2005 at the Kitasato University Hospital were used in this retrospective cohort study. No preoperative chemotherapy and/or radiotherapy case was included. The histological diagnosis was based on the criteria of the World Health Organization/International Association for the Study of Lung Cancer (WHO/IASLC) classification of lung and pleural tumors^29^, and all the adenocarcinomas and squamous cell carcinomas were sub-grouped into well/moderately or poorly differentiated ones. To discriminate the poorly differentiated tumors, squamous cell carcinoma was diagnosed based on keratinization, intercellular bridges and stream-like cell arrangement, while adenocarcinoma was determined by abortive papillary and/or acinar structures and mucous production, although these features were faint. The poorly differentiated tumors completely absent of these hints were diagnosed as large cell carcinoma, and were excluded from the present study. Each case was reassigned for TNM classification and pathological stage on the basis of the new IASLC staging system^30^,^31^. The following clinical and pathological parameters were retrospectively reviewed in each case: age at surgery, gender, smoking habits, histological type, tumor differentiation, p-TNM stage, intratumoral vascular invasion, intratumoral lymphatic invasion, pleural invasion, adjuvant chemotherapy, viability status, and survival time after surgery. Viability status was determined based on whether or not NSCLC-related death occurred, and survival time was defined as the duration from the date of surgery to the date of death or the end of the follow-up. We treated all cases with death caused by other reasons or lost to follow-up as censored cases.

All samples were collected in accordance with the ethical guidelines, written informed consent was received, and this study was approved by the Ethics Committee of Kitasato University School of Medicine. All patients were approached based on approved ethical guidelines, and those who agreed to participate in this study were required to sign consent forms. Patients could refuse entry and discontinue participation at any time. All participants provided written informed consent.

### Immunohistochemical Staining for MUC5B and TTF-1

Three-micrometer-thick sections were made and deparaffinized in xylene, rehydrated in a descending ethanol series, and then treated with 3% hydrogen peroxide for 10 min. After antigen retrieval by autoclaving in 0.01 mol/L citrate buffer (pH 6.0) with 0.1% Tween 20 at 121°C and blocking with 2% normal swine serum (NSS) for 10 min each, the sections were reacted with non-diluted anti-MUC5B monoclonal antibody (hybridoma supernatant) or 200-times-diluted anti-TTF-1 monoclonal antibody (clone; 8G7G3/1, Dako, Glostrup, Denmark) for 2 h at room temperature (RT). After rinsing in Tris-buffered saline (0.01 M Tris-HCl pH 7.5, 150 mM NaCl) three times for 5 min each, the sections were reacted with ChemMATE ENVISION (Dako) for 30 min at RT. The sections were subsequently visualized with Stable DAB solution (Invitrogen; Carlsbad, CA) and counterstained with Mayer's hematoxylin.

### Evaluation of Immunohistochemical Staining

For MUC5B, cytoplasmic staining in tumor cells was considered to be positive. The stainability of bronchial epithelial cells was used as an internal positive control. First, at low magnification, we selected the region of highest expression. MUC5B staining was scored by multiplication of the percentage of positive tumor cells and staining intensity at 400×^32^. The percentage of positive tumor cells was categorized into four groups: 0 = 0%; 1 = 1–25%; 2 = 26–50%; 3 = 51–75%; 4 = 76–100%. Staining intensity was categorized into three groups by comparing the staining intensity of tumor cells with bronchial epithelial cells: 1 (weak) = weaker than epithelial cells; 2 (moderate) = the same as epithelial cells; 3 (strong) = stronger than epithelial cells.

For TTF-1, nuclear staining was considered to be positive. First, at low magnification we selected the region of highest expression. To exclude equivocal reactions, at least 500 cells in five areas were counted for each glass slide. At least moderate intensity in more than 10% of the tumor cells was taken as positive staining^28^. Two investigators (Ryo Nagashio and Yuichi Sato) separately evaluated all the specimens in a blinded manner. Variant cases were reviewed and discussed until a consensus was obtained.

### Statistical Analysis

Continuous variables were presented as the median (range), while numerical variables were given as N (%). The relationships between MUC5B expression and clinicopathological parameters were assessed by Mann–Whitney U-test. Cumulative survival of patients was estimated using the Kaplan-Meier method, and statistical significances of the differences of the survival rate between the MUC5B-high expression (score ≥ 9) and MUC5B-low expression (score < 9) groups, the TTF-1 positive and TTF-1 negative groups, and the four groups which combined both stainabilities were tested using the log-rank test. For the Kaplan-Meier estimate of the survival curves, we truncated the data at a follow-up period of 5 years to avoid the risk of numbers being too small. Those with a survival of more than 5 years were reported as 5 years, and events occurring after the end of the 5-year follow-up period were computed as censored data. Five-year cumulative survival probability was estimated using the life table method with the interval length set at 1 month. Multivariable analysis was performed by employing the Cox proportional hazards regression model to examine the interaction between MUC5B expression and other clinicopathological variables and to estimate the independent prognostic effect of MUC5B on survival by adjusting for confounding factors. There were 57 lung cancer-related deaths in the present study, which allowed a maximum of 5 variables to be included in the multivariable regression model. To avoid over-fitting, all potential confounding factors of MUC5B expression were reduced to one single composite characteristic by applying a propensity score^33^. The conventional P value of 0.05 or less was used to determine the level of statistical significance. All reported P values are two sided. Analyses were performed using StatFlex version 6.0 (Artech Co., Ltd., Osaka, Japan).

## Author Contributions

Project conception and oversight, R.N. and Y.S.; manuscript preparation, R.N., J.U., S.M., M.K., K.Y. and Y.S.; data analysis and bioinformatics, R.N., K.H., B.T. and Y.S.; tissue banking and sera collections, S.R., H.N., S.X.J., K.K., Y.S., N.M. and Y.M.; and pathology review, R.N., H.N., S.X.J., Y.M. and Y.S.

## Supplementary Material

Supplementary Information supplementary information

## Figures and Tables

**Table 1:** Characteristics of the Patients

Characteristics	N = 247
Age	
<65	122 (49.4)
≥65	125 (50.6)
Gender	
Male	154 (62.3)
Female	93 (37.7)
Smoking habit	
No	96 (38.9)
Yes	151 (61.1)
Histology	
AC	198 (80.2)
SCC	49 (19.8)
Tumor differentiation	
Well/moderately	199 (80.6)
Poorly	48 (19.4)
p-TNM stage*	
Stage I	147 (59.5)
Stage II	48 (19.4)
Stage III	52 (21.1)
Receiving adjuvant chemotherapy	
Yes	37 (15.0)
No	210 (85.0)
Vital status	
Alive	146 (59.1)
Lung cancer-related death	76 (30.8)
Other causes of death	17 (6.9)
Unknown	8 (3.2)

Data are presented as No. (%).

AC = adenocarcinoma; p-TNM = pathological TNM; SCC = squamous cell carcinoma.

*Each case was reassigned for pathological stage according to the 7th edition of TNM classification[30, 31].

**Table 2:** Relationship Between MUC5B Expression and Clinicopathological Parameters in Lung Adenocarcinoma

		MUC5B Expression		
Clinicopathological Parameters	Total	Positive Rate (%)	Ave. Score	P Value
Age, y				
<65 y	109	64.2	4.5	0.4723
≥65 y	89	66.3	3.9	
Gender				
Male	108	66.7	4.2	0.7934
Female	90	63.3	4.2	
Smoking habit				
No	93	60.2	4.1	0.4074
Yes	105	69.5	4.4	
Tumor size				
≤5 cm	181	63.5	4.1	0.0693
>5 cm	17	82.4	5.9	
Tumor differentiation				
Well/moderately	168	63.1	3.9	0.0303
Poorly	30	76.7	5.9	
p-TNM stage*				
Stage I	130	59.2	3.7	0.0153
Stage II/III	68	76.5	5.1	
Nodal status				
N0	152	61.8	4.0	0.0733
N1/N2/N3	46	76.1	5.1	
Vascular invasion				
No	106	61.3	3.8	0.1352
Yes	69	70.0	4.9	
Lymphatic invasion				
No	101	66.3	4.2	0.5224
Yes	55	67.3	4.7	
Pleural invasion				
No	136	60.3	3.9	0.0867
Yes	62	75.8	4.8	
Adjuvant chemotherapy				
No	168	66.7	4.4	0.1959
Yes	30	56.7	3.2	

Data are presented as positive rate and average score. See Table 1 legend for expansion of abbreviations.

*Each case was reassigned for pathological stage according to the 7th edition of TNM classification[30, 31].

**Table 3:** Univariable and Multivariable Analysis for the effect of MUC5B Expression on Survival

	Univariable Analysis			Multivariable Analysis		
Factors	HR	95% CI	P Value	HR	95% CI	P Value
MUC5B expression						
High (≥9) vs low (<9)	2.794	1.434–5.444	0.0025	2.658	1.260–5.608	0.0102
Age						
≥65 vs <65	1.046	0.555–1.969	0.8902	n/d	n/d	n/d
Gender						
Male vs female	1.384	0.726–2.639	0.3232	n/d	n/d	n/d
Smoking habit						
No vs Yes	1.525	0.800–2.907	0.1999	n/d	n/d	n/d
Tumor size						
≤5 cm vs >5 cm	6.175	3.000–12.711	<0.0001	3.808	1.537–9.435	0.0039
p-TNM stage						
Stage II/III vs stage I	9.526	4.373–20.749	<0.0001	2.650	1.107–6.344	0.0287
Adjuvant chemotherapy						
No vs yes	6.751	3.585–12.711	<0.0001	4.043	2.213–9.872	0.0001
Tumor differentiation						
Poorly vs well/moderately	3.363	1.701–6.648	0.0005	n/d	n/d	n/d
Vascular invasion						
No vs Yes	10.004	4.146–24.141	<0.0001	3.889	1.465–10.323	0.0064
Lymphatic invasion						
No vs Yes	5.046	2.387–10.668	<0.0001	n/d	n/d	n/d
Pleural invasion						
No vs Yes	2.935	1.563–5.511	0.0008	n/d	n/d	n/d

n/d: not done.

**Table 4:** Relationship Between TTF-1 Expression and Clinicopathological Parameters in Lung Adenocarcinoma

		TTF-1 Expression		
Clinicopathological Parameters	Total	Positive (n = 161)	Negative (n = 37)	P Value
Age, y				
<65 y	109	92 (84.4)	17 (15.6)	0.2170
≥65 y	89	69 (77.5)	20 (22.5)	
Gender				
Male	108	83 (76.9)	25 (23.1)	0.0777
Female	90	78 (86.7)	12 (13.3)	
Smoking habit				
No	93	84 (90.3)	9 (9.7)	0.0025
Yes	105	77 (73.3)	28 (26.7)	
Tumor size				
≤5 cm	181	154 (85.1)	27 (14.9)	<0.0001
>5 cm	17	7 (41.2)	10 (58.8)	
Tumor differentiation				
Well/moderately	168	143 (85.1)	25 (14.9)	0.0011
Poorly	30	18 (60.0)	12 (30.0)	
p-TNM stage*				
Stage I	130	113 (86.9)	17 (13.1)	0.0051
Stage II/III	68	48 (70.6)	20 (29.4)	
Nodal status				
N0	152	121 (79.6)	31 (20.4)	0.2624
N1/N2/N3	46	40 (87.0)	6 (13.0)	
Vascular invasion				
No	106	89 (84.0)	17 (16.0)	0.4715
Yes	69	55 (79.7)	14 (20.3)	
Lymphatic invasion				
No	101	83 (82.2)	18 (17.8)	0.5999
Yes	55	47 (85.5)	8 (14.5)	
Pleural invasion				
No	136	115 (84.6)	21 (15.4)	0.0827
Yes	62	46 (74.2)	16 (25.8)	
Adjuvant chemotherapy				
No	168	135 (80.4)	33 (19.6)	0.4141
Yes	30	26 (86.7)	4 (13.3)	

Data are presented as positive rate and average staining score. See Table 1 legend for expansion of abbreviations.

*Each case was reassigned for pathological stage according to the 7th edition of TNM classification[30, 31].
