# Supplementary material for: Diagnostic and Prognostic Significances of MUC5B and TTF-1 Expressions in Resected Non-Small Cell Lung Cancer
Source: Sci Rep. 2015 Mar 3;5:8649. doi: 10.1038/srep08649 (PMC4346793; doi:10.1038/srep08649)
Supplement: Supplementary Information [file srep08649-s1.pdf]

Supplementary information

Diagnostic and Prognostic Significances of MUC5B and TTF-1 Expressions in Resected Non-Small Cell Lung Cancer

Ryo Nagashio, Junpei Ueda, Shinichiro Ryuge, Hiroyasu Nakashima, Shi-Xu Jiang, Makoto Kobayashi, Kengo Yanagita, Ken Katono, Yukitoshi Satoh, Noriyuki Masuda, Yoshiki Murakumo, Kazuo Hachimura, Yuichi Sato

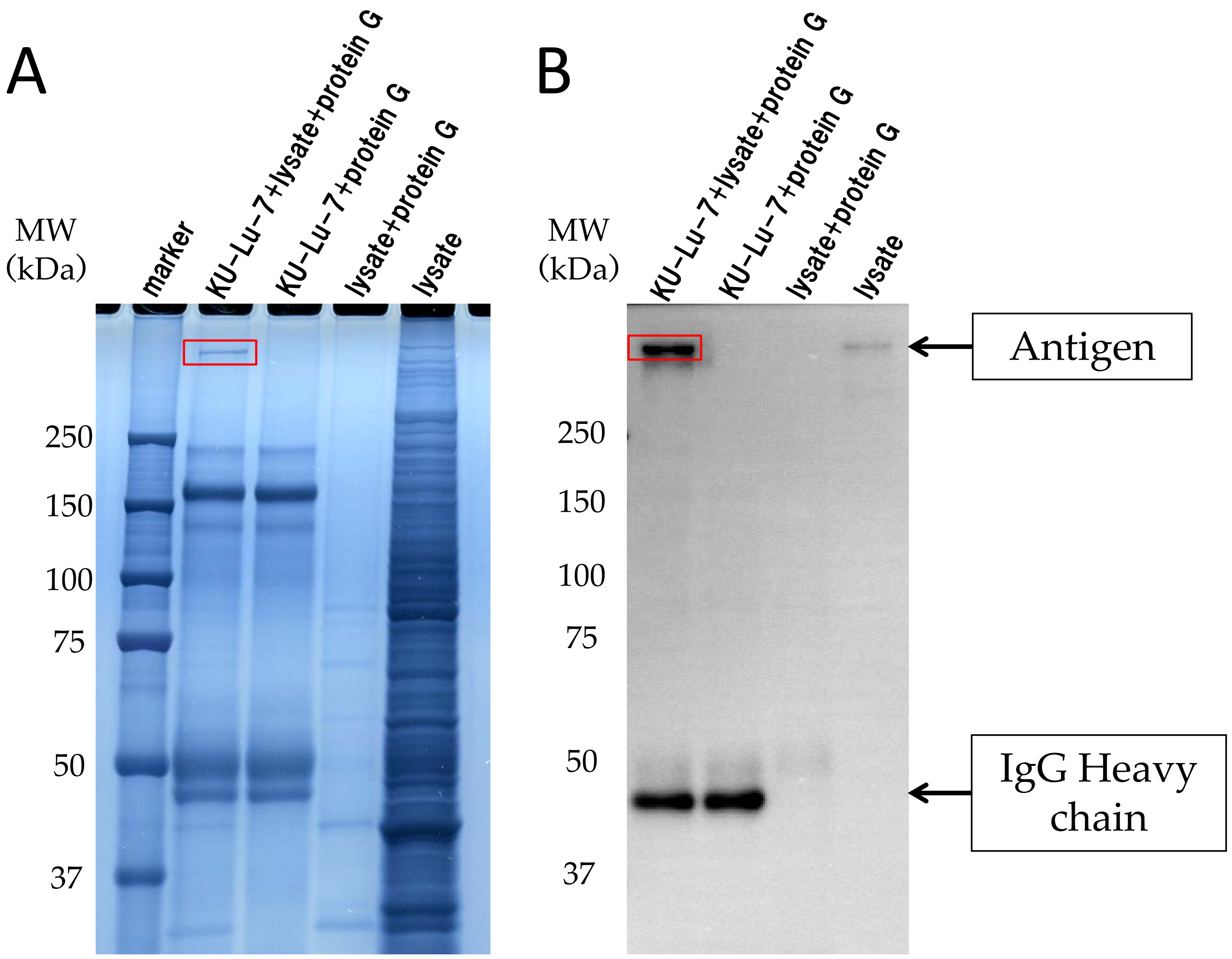

Supplementary Figure 1.
Identification of KU-Lu-7 monoclonal antibody by immunoprecipitation and mass spectrometry. The proteins immunoprecipitated with KU-Lu-7 antibody were separated with SDS-PAGE and the gel was stained with Zn-staining (A). [lane 1: A549 lysate combined with KU-Lu-7 antibody, lane 2: KU-Lu-7 antibody combined with protein G, lane 3:A549 lysate combined with protein G, lane 4: A549 lysate]. Immunoblot analysis using KU-Lu-7 hybridoma supernatant as the first antibody (B). Lanes 2 to 3 are negative controls, and the antigen immunoprecipitated with KU-Lu-7 antibody was detected in lane 1 (red rectangle). Based on MALDI TOF/TOF-MS analysis, the KU-Lu-7 antibody recognized MUC5B.
